# Late-onset myocardial infarction and autoimmune haemolytic anaemia in a COVID-19 patient without respiratory symptoms, concomitant with a paradoxical increase in inflammatory markers: a case report

**DOI:** 10.1186/s13256-020-02595-3

**Published:** 2020-12-18

**Authors:** Maria Chiara Pelle, Bruno Tassone, Marco Ricchio, Maria Mazzitelli, Chiara Davoli, Giada Procopio, Anna Cancelliere, Valentina La Gamba, Elena Lio, Giovanni Matera, Angela Quirino, Giorgio Settimo Barreca, Enrico Maria Trecarichi, Carlo Torti, IDTM UMG COVID-19 Group, Maria Chiara Pelle, Bruno Tassone, Marco Ricchio, Maria Mazzitelli, Chiara Davoli, Giada Procopio, Anna Cancelliere, Valentina La Gamba, Elena Lio, Giovanni Matera, Angela Quirino, Giorgio Settimo Barreca, Enrico Maria Trecarichi, Carlo Torti, Eugenio Arrighi, Bernardo Bertucci, Maria Teresa Busceti, Claudio Carallo, Francesco Saverio Costanzo, Adele De Francesco, Paolo Fusco, Luigia Gallo, Aida Giancotti, Amerigo Giudice, Giuseppe Greco, Domenico Laganà, Angelo Lamberti, Maria Carla Liberto, Rosaria Lionello, Nadia Marascio, Giuseppina Marrazzo, Maria Petullà, Graziella Perri, Vincenzo Scaglione, Francesca Serapide, Ciro Indolfi, Federico Longhini, Andrea Bruni, Eugenio Garofalo

**Affiliations:** 1 Infectious and Tropical Disease Unit, Department of Medical and Surgical Sciences, “Magna Graecia” University of Catanzaro, Viale Europa, 88100 Catanzaro, Italy; 2 Department of Health Sciences, Unit of Clinical Microbiology, University “Magna Graecia”, Catanzaro, Italy; 3 “Mater Domini” teaching hospital, Unit of Clinical Microbiology, Catanzaro, Italy

**Keywords:** COVID-19, Inflammation, Cardiovascular disease, AIHA, Anaemia, IL-6

## Abstract

**Background:**

In December 2019, a new coronavirus (named severe acute respiratory syndrome coronavirus 2, SARS-CoV-2) spread from China, causing a pandemic in a very short time. The main clinical presentation of SARS-CoV-2 infection (COVID-19, coronavirus disease-2019) is pneumonia, but several cardiovascular complications may also occur (e.g., acute coronary syndromes, pulmonary embolism, stroke, arrhythmias, heart failure and cardiogenic shock). Direct or indirect mechanisms induced by SARS-CoV-2 could be implicated in the pathogenesis of these events.

**Case presentation:**

We report herein the third case of COVID-19 autoimmune haemolytic anaemia (AIHA) reported so far, which occurredwithout any other possible explanations in a Caucasian patient. The patient also suffered from ST-elevation myocardial injury.

**Conclusions:**

Both complications occurred quite late after COVID-19 diagnosis and were probably precipitated by systemic inflammation, as indicated by a significant delayed increase in inflammatory markers, including interleukin-6 (IL-6).

## Background

The main serious clinical presentation of COVID-19 is pneumonia, but several cardiovascular complications may also occur (e.g., acute coronary syndromes, pulmonary embolism, stroke, arrhythmias, heart failure and cardiogenic shock) [1]. Moreover, there are few studies describing AIHA during COVID-19 [2]. We report herein the third case of COVID-19 autoimmune haemolytic anaemia, which occurred after an ST-elevation myocardial injury in a patient without any respiratory symptoms.

## Clinical case presentation

**Figure 1.**
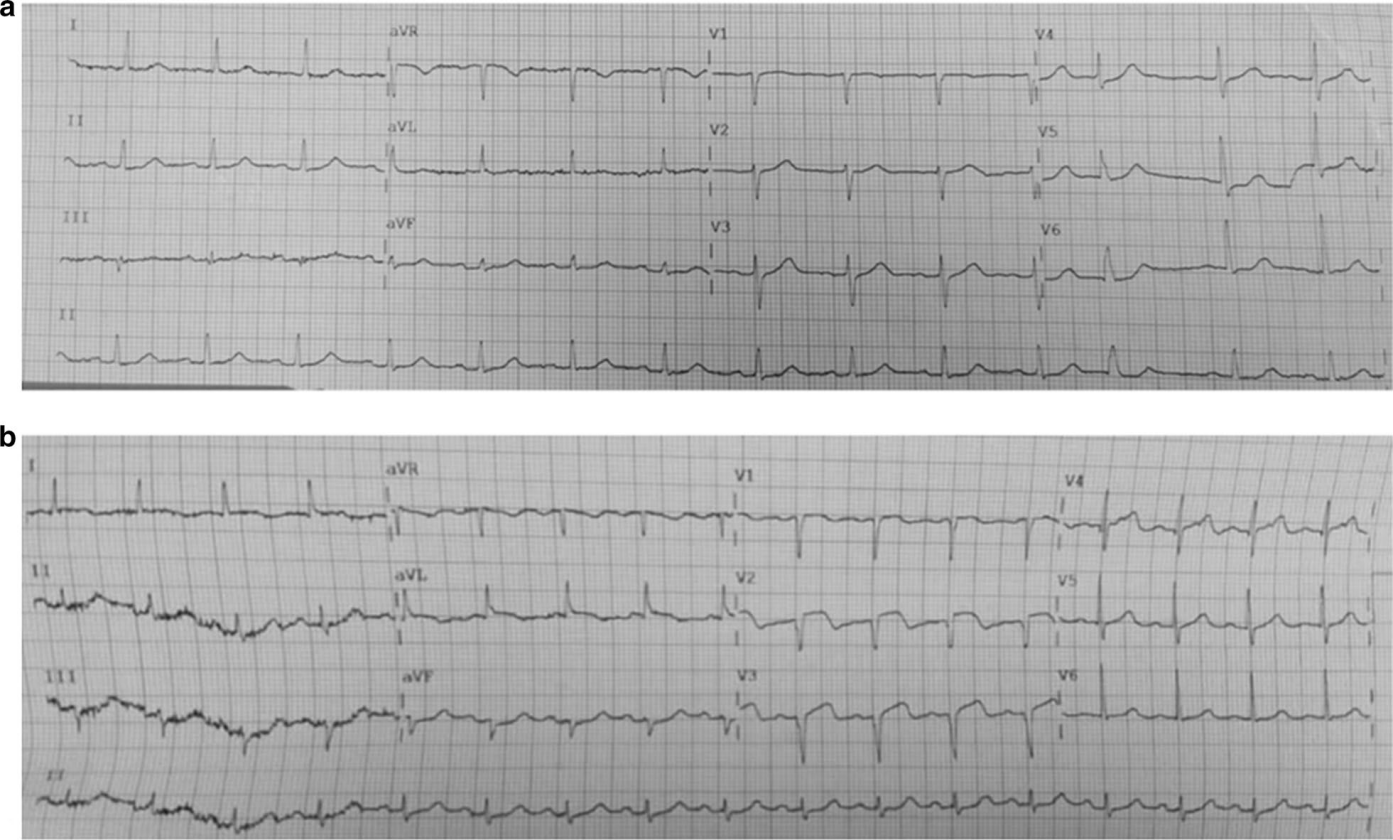
Baseline electrocardiogram (**a**) and the occurrence of myocardial infarction (**b**)

An 86-year-old Caucasian woman suffering from hypertension and anxiety-depressive syndrome was admitted to our Infectious and Tropical Disease Unit on April 1st, 2020. She tested positive for severe acute respiratory syndrome coronavirus 2 (SARS-CoV-2) by nasopharyngeal swab on March 26th, 2020, and did not report any symptoms other than nausea. Physical and neurological examinations on admission were normal, blood pressure was 120/85 mmHg, heart rate was 92 beats per minute, and temperature was 36.8°C. She resided in a long-term care facility and was on treatment with trazodone 60 mg/ml 3 drops daily and delorazepam 1 mg/ml 5 drops daily for an anxiety syndrome and furosemide 25 mg 1 tablet three times a week for high blood pressure. She was unmarried, had no children, was a housewife and lived in an urban area. She did not smoke and did not consume alcohol. In Table 1, we report the main laboratory findings at diagnosis and during the course of her hospital stay. The baseline twelve-lead electrocardiogram was normal (Fig 1a). Although she was asymptomatic, we performed a chest X-ray, which showed a“thickening of the interstitial design, in particular at the bases”. Due to this finding, antiviral treatment with hydroxychloroquine and azithromycin was started according to a protocol reported elsewhere and clinical guidelines [3, 4]. Due to hypomobility, thromboprophylaxis with enoxaparin 4000 IU once daily was also started (Padua score = 4) [5]. During hospitalization, the patient did not need any oxygen support. Swabs were persistently positive over time until they became negative on May, 15th, 2020. Therefore, positivity lasted for a total of 54 days. Despite a favourable clinical course without any respiratory symptoms, c-reactive protein (CRP) and IL-6 increased progressively, with IL-6 reaching a peak of 165 pg/ml, concomitant with the occurrence of the complications described below (in particular AIHA).

**Table 1.** Haemato-biochemical values at hospitalization and at the occurrence of complications.

Exam	Unit	Normality range	April 1st 2020	May 8th 2020	May 13th 2020	May 26th 2020
Red blood cells	x10^6^/µl	4.2-5.4	3.49	3.38	2.91	3.50
Haemoglobin	g/dl	12-16	10.6	10	8.3	10.4
Haematocrit	%	37-47	31.7	30.1	25.8	30.8
MCV	fl	81-99	90.9	89	88.7	87.9
White blood cells	x10^3^/µl	5.2-12.4	3.59	6.69	4.94	9.69
Neutrophils	%	40-74	70.3	71.3	85.2	85.1
Neutrophils	x10^3^/ µl	1.9-8	2.52	4.77	4.21	8.25
Lymphocytes	%	19-48	19.8	16.1	7.7	7.9
Lymphocytes	x10^3^/ µl	0.9-5.2	0.71	1.08	0.38	0.77
Platelets	x10^3^/µl	130-400	178	188	211	327
Ferritin	ng/ml	30-400	388	501	548	719
Creatinine	mg/dl	0.7-1.2	0.74	0.67	0.62	0.47
Total cholesterol	mg/dl	0-199	154	151	110	148
HDL-cholesterol	mg/dl	>35	45	60	49	66
LDL-cholesterol	mg/dl	<130	94	76	47	59
Triglyceride	mg/dl	<150	98	106	87	110
Total bilirubin	mg/dl	<1.1	0.68	0.94	1.01	1.15
Direct bilirubin	mg/dl	<0.3	0.07	0.29	0.49	0.47
Indirect bilirubin	mg/dl		0.61	0.65	0.52	0.68
ALT (GPT)	IU/l	<41	10	12	17	14
AST (GOT)	IU/l	<38	42	37	29	20
LDH	IU/l	240-480	794	773	598	599
CPK	mmol/l	20-190	91	187	376	112
PT	%	70-120	113	72	59	35
PT	sec		13	14	15	23
INR	I.N.R.		1.01	1.31	1.41	2.23
APTT	sec	25.9-36.6	29	31	40	41
D-dimer	mg/l	0.0-0.55	0.46	0.57	1.38	0.37
Fibrinogen	mg/dl	200-400	461	373	569	395
Sodium	mmol/l	136-145	134	126	140	129
Potassium	mmol/l	3.5-5.1	4.4	3.6	3.1	3.8
IL-6	pg/mL	<7.00	22.98	16.59	164.80	--
CRP	mg/l	0.0-5.0	7	15.6	--	45
PCT	ng/ml	<0.05	<0.05	<0.05	0.08	<0.05
Haptoglobin	gr/l	0.3-2.0	--	--	1.2	--
Reticulocytes	%	0.5-2.5	--	--	1.64	--

MCV: Mean Corpuscular Volume; HDL: High-Density Lipoprotein; LDL: Low-Density Lipoprotein; ALT: Alanine aminotransferase; GPT: Glutammate Pyruvate Transaminase; AST: Aspartate Transaminase; GOT: Glutamic Oxaloacetic Transaminase; LDH: Lactate DeHydrogenase; CPK: Creatine PhosphoKinase; PT: Prothrombin Time; INR: International Normalized Ratio; APTT: partial thromboplastin time; IL-6: Interleukin-6; CRP: C-Reactive Protein; PCT: procalcitonin

On April 11th, for abdominal and right shoulder pain, the patient underwent a computerized tomography (CT) scan, which demonstrated gallbladder lithiasis and areas of ground glass opacity in the lungs. A few days later, due to an accidental fall, she underwent a cerebral CT scan, which showed evidence of only chronic cerebral vasculopathy. As a precaution, due to the traumatic event, given the improvement in patient mobility, enoxaparin was stopped.

On May 8^th^, the patient developed paraesthesia of the left arm; therefore, an electrocardiogram was performed and showed evidence of an ST-elevation myocardial injury (Fig 1b). Cardiac biomarkers were elevated: the highest value of high-sensitivity troponin T was 268.3 ng/L, myoglobin was 128 ng/ml and creatine kinase-MB was 12.4 ng/ml, with a progressive reduction over the following days. Taking into account advanced age and patient fragility, cardiologists advised against a more aggressive approach, limiting medical therapy to intravenous acetylsalicylic acid 125 mg on the first day, followed by oral administration100 mg/day; oral clopidogrel 75 mg 4 tablets on the first day, followed by 75 mg/day; subcutaneous enoxaparin 4000 IU twice daily for 7 days; oral ramipril 5 mg once daily and oral atorvastatin 40 mg once daily. The patient was then transferred to the intensive care unit (ICU) for clinical and instrumental monitoring. An echocardiogram was performed, showing a hypertrophic left ventricle with normal systolic function (ejection fraction, EF, 55%), a normokinetic left ventricle apex, and normal right sections. The level of NT-proBNP was 5404 pg/ml. Moreover, for marked hypotension, therapy with norepinephrine (0.2 γ/kg/min) was prescribed.

After three days in the Intensive Care Unit (ICU), the patient’s clinical condition improved; therefore, she was transferred again to our unit. A reduction in haemoglobin of 2 grams was found on May 13^th^, 2020, compared to a value of 10 grams/dl 5 days before. To understand the aetiology of anaemia, ferritin, sideraemia, vitamin B12, folic acid were measured, but only folic acid was low. Haemophagocytic lymphohistiocytosis syndrome was excluded [6]. Direct and indirect Coombs tests were performed as well, and the direct Coombs test was highly positive (i.e., 4+) for immunoglobulin (IgG). The consulting haematologist performed a peripheral blood smear, which indicated “anisopoikilocytosis of erythrocytes, several acanthocytes, some schistocytes”. After consideration of this result, the consulting haematologist decided against a diagnosis of a lymphoproliferative disorder, there by suggesting the avoidance of bone biopsy and possible contraindications, such as the need for antiplatelet therapy for recent myocardial infarction. Since antibodies (IgG) against *Cytomegalovirus* (CMV) were positive, although IgM were negative, to exclude a possible role of *Cytomegaloviru*s reactivation in the genesis of AIHA, CMV, Deoxyribo Nucleic Acid (DNA) was tested in serum using real time Polymerase Chain Reaction (PCR) (CMV Elite MGB® Kit, Elitech Group, Italia). With this method, CMV DNA was undetectable. Also, IgG for *Parvovirus B-19* were positive, IgM were negative, and *Parvovirus B19* DNA was undetectable in serum by real time PCR. Therefore, diagnosis of these two active infections was excluded.

High-dose corticosteroid treatment (prednisolone 0.8 mg/kg) was started, although anaemia was only mild because the patient had worsening asthenia and dyspnoea, while transfusion would have been dangerous due to the presence of IgG autoantibodies. Rapid improvement in haemoglobin levels was obtained (Table 1). On May, 27th 2020, echocardiogram was performed, which showed a hypokinetic cardiac apex and mild mitral regurgitation. The patient’s clinical condition improved; therefore, the patient was discharged on July 3rd 2020 in good conditions and then hosted in a long-term care facility. The patient did not carry out follow-up visits.

## Discussion

Herein, we report a case of AIHA occurring during COVID-19 concomitant with myocardial infarction. These complications occurred when IL-6 peaked at a high plasma concentration late during the course of COVID-19. To the best of our knowledge, this is the first report showing late-onset AIHA occurring during COVID-19 concomitant with myocardial infarction, another complication that may be due to a hyperinflammatory status. Moreover, AIHA occurred in a patient who did not have any pre-existing haematological diseases or predisposing conditions, while in most similar cases reported so far, patients suffered from neoplastic or lymphoproliferative disorders.

Several studies highlighted that cardiovascular complications may occur in patients with SARS-CoV-2 infection. Indeed, SARS-CoV-2 can cause direct myocardial damage. In fact, the virus uses the angiotensin-enzyme-2 (ACE-2) host protein as a coreceptor to enter human cells, and this receptor is overexpressed in the heart and vascular system [7, 8]. Additionally, the cardiovascular system may be affected by systemic inflammation, causing organ damage as an effect of a cytokine storm [9, 10]. In addition, systemic inflammation can facilitate the rupture of pre-existing plaques, resulting in acute myocardial infarction. Concomitant causes of myocardial damage can be the altered ratio of myocardial demand-supply and increased cardiometabolic demand and hypoxia induced by lung disease [1]. Last, pre-existing cardiovascular diseases may have an impact on inducing heart injury [10, 11]. Our patient had hypertension and advanced age as risk factors. A cerebral CT scan evidenced chronic cerebral vasculopathy, possibly indicating a polyvascular atherosclerosis. Additionally, ongoing inflammation could have facilitated the rupture of pre-existing plaques. Therefore, myocardial infarction was somehow predictable. Even though the actual incidence of ST-segment elevation myocardial infarction in COVID-19 (as occurred in our patient) has not been described [12], the prevalence of acute myocardial injury in COVID-19 patients was reported to range from 7.2% to 17% [9, 10, 13]. Given the high incidence of myocardial infarction, especially in patients with underlying cardiovascular risks (as was the case in the patient presented herein), we should probably not have stopped enoxaparin when the accidental fall occurred, notwithstanding the risk of haemorrhagic events [14]. This consideration further confirms the need for improved algorithms and guidelines for the use of heparin in COVID-19 patients.

Immediately after myocardial infarction, our patient developed AIHA. This is an uncommon disorder characterized by the production of antibodies directed against self-red blood cells, which are destroyed through complement-mediated mechanisms and the reticuloendothelial system. Concurrent lymphoproliferative diseases, autoimmune diseases, *Mycoplasma* and infections due to several viruses, such as Epstein-Barr virus, *Coxsackie*, *Cytomegalovirus*, hepatitis C virus, and *Parvovirus B19*, may trigger AIHA. A possible aetiological mechanism is molecular mimicry, since these viruses possess structures that mimic normal host self-proteins, so the immune system activated against the pathogen may cross-react with self-antigens [15, 16]. Since we excluded possible alternative causes, we conclude that persistent inflammation due to SARS-CoV-2 infection was responsible for AIHA.

There are few studies describing AIHA during COVID-19. In particular, Lazarian *et al.* [17] described 7 cases of AIHA associated with COVID-19. These cases occurred during the course of the disease earlier (i.e., a median of 9 days) after diagnosis, and in only one case did the patient not suffer from neoplastic diseases (mainly lymphoproliferative disorders), which may explain the occurrence of AIHA. In contrast, our patient suffered from AIHA later on during the course of COVID-19, and no other obvious explanations were found for AIHA. Lopez *et al.* [18] described another case of direct Coombs test positive for IgG and C3 during COVID-19, but this patient had a medical history of congenital thrombocytopenia. Last, Hindilerden *et al.* [2] reported another case of AIHA during COVİD-19 in the absence of associated underlying disorders, showing a direct Coombs test positive for IgG and C3d. In conclusion, we present herein the third case of AIHA in a patient without underlying neoplastic or haematological concomitant disorders. Similar to the cases previously reported, our patient showed elevated markers of inflammation (i.e., ferritin, CRP, and D-dimers) at the time of AIHA diagnosis. Interestingly, at that point, IL-6 peaked at 165 pg/ml, starting from 22 pg/ml at the time of admission. We feel that hyperinflammation was pathogenically linked to both myocardial infarction and AIHA. Indeed, in our cohort, IL-6 appeared to be an independent predictor of death, together with elevated CRP [19].

With regard to therapy, corticosteroids are recommended as the first-line treatment for AIHA [20]. In line with this recommendation, our patient was treated with high-dose prednisolone (0.8 mg/kg/day), while in the other case series reported so far, intravenous immunoglobulin at 1 g/kg/day was used first, with suboptimal response. Only when corticosteroids were prescribed was a significant response obtained, and in only one case was rituximab used after a poor response to corticosteroids [16, 17]. Therefore, our case appears to support current guidelines [20] indicating that corticosteroids should be the first-line treatment in AIHA, even when this complication occurs after COVID-19. However, as highlighted in the clinical case presentation, corticosteroid treatment was prescribed in our patient not withstanding mild anaemia because the patient was symptomatic, probably for concomitant myocardial damage, so further studies are necessary to validate this treatment in patients with more severe anaemia.

## Conclusion

Although the underlying mechanisms are not well known, we confirm that SARS-CoV-2 infection may trigger AIHA, as well as other complications, such as myocardial infarction, possibly related to cytokine storm and hyperinflammation. This is further supported by the concomitant increase in inflammatory markers such as IL-6 in our patient. Moreover, our case highlights the need for laboratory and clinical surveillance of patients hospitalized for COVID-19 for a prompt diagnosis of late-onset complications, especially when markers such as IL-6 do not return to normal or paradoxically increase during the course of an otherwise benign disease.

## Data Availability

All data generated or analysed during this study are included in this article.

## Abbreviations

SARS-CoV-2: Severe acute respiratory syndrome coronavirus 2
COVID-19: Coronavirus disease-2019
AIHA: Autoimmune haemolytic anaemia
CRP: C-reactive protein; IL-6: interleukin-6
CT: Computed tomography
ICU: Intensive care unit
